# Studies of the in vitro anticancer, antimicrobial and antioxidant potentials of selected Yemeni medicinal plants from the island Soqotra

**DOI:** 10.1186/1472-6882-9-7

**Published:** 2009-03-25

**Authors:** Ramzi A Mothana, Ulrike Lindequist, Renate Gruenert, Patrick J Bednarski

**Affiliations:** 1 Department of Pharmacognosy, Faculty of Pharmacy, Sana'a-University, PO Box 33039, Sana'a, Yemen; 2 Department of Pharmaceutical Biology, Institute of Pharmacy, Ernst-Moritz-Arndt-University, Greifswald, FL-Jahnstrasse 15a, D-17487, Greifswald, Germany; 3 Department of Pharmaceutical and Medicinal Chemistry, Institute of Pharmacy, Ernst-Moritz-Arndt-University, Greifswald, FL-Jahnstrasse 17, D-17487, Greifswald, Germany

## Abstract

**Background:**

Recent years have witnessed that there is a revival of interest in drug discovery from medicinal plants for the maintenance of health in all parts of the world. The aim of this work was to investigate 26 plants belonging to 17 families collected from a unique place in Yemen (Soqotra Island) for their in vitro anticancer, antimicrobial and antioxidant activities.

**Methods:**

The 26 plants were extracted with methanol and hot water to yield 52 extracts. Evaluation for in vitro anticancer activity was done against three human cancer cell lines (A-427, 5637 and MCF-7) by using an established microtiter plate assay based on cellular staining with crystal violet. Antimicrobial activity was tested against three Gram-positive bacteria, two Gram-negative bacteria, one yeast species and three multiresistant *Staphylococcus *strains by using an agar diffusion method and the determination of MIC against three Gram-positive bacteria with the broth micro-dilution assay. Antioxidant activity was investigated by measuring the scavenging activity of the DPPH radical. Moreover, a phytochemical screening of the methanolic extracts was done.

**Results:**

Notable cancer cell growth inhibition was observed for extracts from *Ballochia atro-virgata*, *Eureiandra balfourii *and *Hypoestes pubescens*, with IC_50 _values ranging between 0.8 and 8.2 μg/ml. The methanol extracts of *Acanthospermum hispidum*, *Boswellia dioscorides*, *Boswellia socotrana*, *Commiphora ornifolia *and *Euphorbia socotrana *also showed noticeable antiproliferative potency with IC_50 _values < 50 μg/ml. The greatest antimicrobial activity was exhibited by extracts from *Acacia pennivenia*, *Boswellia *dioscorides, *Boswellia socotrana*, *Commiphora ornifolia*, *Euclea divinorum*, *Euphorbia socotrana*, *Leucas samhaensis*, *Leucas virgata*, *Rhus thyrsiflora*, and *Teucrium sokotranum *with inhibition zones > 15 mm and MIC values ≤ 250 μg/ml. In addition, the methanolic extracts of *Acacia pennivenia*, *Boswellia dioscorides*, *Boswellia socotrana *and *Commiphora ornifolia *showed good antioxidant potential at low concentrations (more than 80% at 50 μg/ml).

**Conclusion:**

Our results show once again that medicinal plants can be promising sources of natural products with potential anticancer, antimicrobial and antioxidative activity. The results will guide the selection of some plant species for further pharmacological and phytochemical investigations.

## Background

The worldwide use of natural products including medicinal plants has become more and more important in primary health care especially in developing countries. Many pharmacognostical and pharmacological investigations are carried out to identify new drugs or to find new lead structures for the development of novel therapeutic agents for the treatment of human diseases such as cancer and infectious diseases [1]. In developing countries and particularly in Yemen, a large segment of the population still rely on folk medicine to treat serious diseases including infections, cancers and different types of inflammations.

The Soqotra Archipelago in Yemen has long been a land of mystery. Over the centuries travelers returned from the Indian Ocean isles with bizarre tales of trees yielding dragon's blood and cucumbers, forests of frankincense and towering pinnacles shrouded in mist [2]. Soqotra is considered the "jewel" of biodiversity in the Arabian Sea. The long geological isolation of the Soqotra archipelago and its fierce heat and many droughts have combined to create a unique and spectacular endemic flora. Surveys have revealed that more than a third of the 800 or so plant species of Soqotra are found nowhere else [2]. Botanists rank the flora of Soqotra among the ten most endangered island flora in the world.

Currently, there is insufficient scientific research on the plants from Soqotra. In previous studies we described investigations of some endemic and non-endemic plants from the island Soqotra for their antimicrobial [3], antiviral [4], enzyme-inhibitory [5] and anticancer activity [6]. Since a literature search indicated the absence of further information regarding biological and phytochemical investigations of plants from Soqotra, this study was carried out as a part of our continued exploration of Yemeni medicinal plants for interesting biological activities. Thus, the main aim of the present project was to carry out a phytochemical and biological investigation on selected plants from the island of Soqotra, especially on those that are endemic and those that find use in the traditional medicine. In this study, 26 plants belonging to 17 families were collected for evaluation of their cytotoxic, antimicrobial and antioxidant activities and the main chemical contents.

## Methods

### Plant materials

The plants (Table 1) were collected from different locations of the island of Soqotra in the Winter of 2006 and identified at the Pharmacognosy Department, Faculty of Pharmacy, Sana'a University. Part of the identification of the investigated plants was done by Dr. Anthony G. Miller from the Royal Botanic Garden at Edinburgh, UK. Voucher specimens were deposited at the Pharmacognosy Department, Faculty of Pharmacy, Sana'a University.

**Table 1:** List of plants screened in the study.

Plant	Voucher specimen no.	Family	Part tested	Traditional uses^a^
Acacia pennivenia* Schweinf.	Mo-Sq28	Mimosaceae	L	As a paste around the breast for women with mastitis
*Acanthospermum hispidum *DC.	Mo-Sq10	Astraceae	L	Unknown
Acridocarpus socotranus* Oliv.	Mo-Sq16	Malpighiaceae	L, S	Headaches, paralysis and muscle or tendon pain
Aloe perryi* Baker	Mo-Sq9	Aloaceae	R	For eye and stomach problems, constipation and malaria
Ballochia atro-virgata* Balf.f.	Mo-Sq15	Acanthaceae	S, L	Unknown
Blepharis spiculifolia* Balf.f.	Mo-Sq13	Acanthaceae	L, S	Unknown
Boswellia dioscorides* Thulin & Gifri	Mo-Sq26	Burseraceae	B	Common cold, bronchitis, asthma and rheumatism
Boswellia socotrana* Balf.f.	Mo-Sq24	Burseraceae	B	As *Boswellia dioscorides*
*Capparis cartilaginea *Decne.	Mo-Sq8	Capparaceae	L	To treat itching, shortness of breath, head cold and for tumors
Commiphora ornifolia* (Balf.f.) Gillett	Mo-Sq23	Burseraceae	B	Antiseptic, diarrhea, dysentery and emmenagogue
Corchorus erodioides* Balf.f.	Mo-Sq27	Tiliaceae	L, F	Diuretic and urinary tract infections
Croton socotranus* Balf.f.	Mo-Sq4	Euphorbiaceae	L, T	For wounds
*Euclea divinorum *Hiern	Mo-Sq1	Ebenaceae	R	For oral care, tooth ache, fungal diseases, sores, wounds and abscesses
Euphorbia socotrana* Balf.f.	Mo-Sq5	Euphorbiaceae	L	For skin diseases and wounds
Eureiandra balfourii* Cogn.	Mo-Sq3	Cucurbitaceae	L	Unknown
*Ficus cordata *Thunb.	Mo-Sq4	Moraceae	L	Antiseptic and for ulcers and wounds
*Glossonema revoili *Franch.	Mo-Sq29	Asclepiadaceae	L, F	Increase milk production in breastfeeding women
Hibiscus noli-tangere* A.G.Mill.	Mo-Sq30	Malvaceae	L, R	For snake bite and fever in children
Hypoestes pubescens* Balf.f.	Mo-Sq12	Acanthaceae	L	Fungal skin diseases and scabies
Lannea transulta* (Balf.f.) Radcl.-Sm.	Mo-Sq17	Anacardiaceae	L	Haemostatic for wounds, and sores and abrasions
Leucas samhaensis* Cortes-Burns & A.G.Mill.	Mo-Sq23	Labiatae	L	For cough and cold
Leucas virgata* Balf.f.	Mo-Sq21	Labiatae	L	For persons with heartburn and indigestion and stomach problems
Lycium sokotranum* Wagner & Vierh.	Mo-Sq20	Solanaceae	L, S	For stomach ailments and encourage the wound healing
*Maerua angolensis *DC.	Mo-Sq7	Capparaceae	L	To treat fever, aches and general malaise
Rhus thyrsiflora* Balf.f.	Mo-Sq18	Anacardiaceae	T, L	To treat anorexia, general tonic and for painful joints
Teucrium sokotranum* Vierh.	Mo-Sq22	Labiatae	F, L	As flavoring agent and for indigestion

* endemic plant, B: Bark, F: Flower, L: Leaves, R: Roots or rhizomes, S: Stems, T: Fruits

^a ^Most of the information of traditional use has been taken from [2] and native people.

### Extraction of plant materials

The air-dried and powdered plant materials (10 g of each) were extracted with 400 ml methanol (CH_3_OH) by Soxhlet extraction for 8 hours. The residue was dried over night and then extracted with 250 ml water (H_2_O) by using a shaking water-bath at 70°C for 2 hours. The obtained methanolic and water extracts were filtered and evaporated by using a rotary evaporator and freeze dryer. The dried extracts were stored at -20°C until used.

### Determination of antimicrobial activity

#### Test organisms

The following microorganisms were used as test organisms in the screening: 3 Gram-positive strains namely, *Staphylococcus aureus *(ATCC 6538), *Bacillus subtilis *(ATCC 6059), *Micrococuss flavus *(SBUG 16), 2 Gram-negative strains namely, *Escherichia coli *(ATCC 11229), *Pseudomonas aeruginosa *(ATCC 27853) and one fungal strain *Candida maltosa *(SBUG 700). In addition, three multiresistant *Staphylococcus *strains namely, *Staphylococcus epidermidis *847, *Staphylococcus haemolyticus *535, and *Staphylococcus aureus *North German Epidemic Strain (supply from the Institute of Hygiene of Mecklenburg-Vorpommern, Greifswald, Germany) were also employed as test organisms. Stock cultures were maintained at 4°C on slopes of nutrient agar. The cultures were diluted to achieve optical densities corresponding to 2.0 × 10^6 ^colony forming units (CFU/ml) for bacterial and 2.0 × 10^5 ^spore/ml for fungal strains.

#### Antimicrobial assay

The disc-diffusion assay [7] was used to determine the antimicrobial potential of the investigated extracts. Nutrient agar (OXOID LTD, Basingstoke, Hampshire, England) was prepared by dissolving of 27 g/l in water. The sterile nutrient agar was inoculated with microbial cells (200 μl of microbial cell suspension in 20 ml agar medium) and poured into sterile petri dishes. Sterile filter paper discs of 6 mm diameter (Schleicher and Schuell, ref. No. 10321260, lot. DG0274-1) were impregnated with 20 μl of the extract solution (equivalent to 4 mg of the dried extract). The paper discs were dried and placed on the surface of the inoculated agar plates. Plates were kept for 2 hours in refrigerator to enable prediffusion of the extracts into the agar. Then the plates were incubated overnight (18 hours) at 37°C. In contrast, *M. flavus *was incubated at room temperature for 48 h and *C. maltosa *was incubated at 28°C for 48 h. Ampicillin, gentamicin and amphotericin B were used as positive control. Negative controls were performed with paper discs loaded with 20 μl of organic solvents (methanol and 5% ethanol) and dried. At the end of the incubation period the antimicobiral activity was evaluated by measuring the inhibition zones (diameter of inhibition zone plus diameter of the disc). An inhibition zone of 15 mm or more was considered as high antibacterial activity.

#### Broth micro-dilution assay for minimum inhibitory concentrations (MIC)

The broth micro-dilution method described by [8] with modifications was used to determine the MIC of extracts against the three standard Gram-positive strains. With sterile round-bottom 96-well plates, duplicate two-fold serial dilutions of extract (100 μl/well) were prepared in the appropriate broth containing 5% (v/v) DMSO to produce a concentration range of 2000 to 15.6 μg of extract/ml. Two-fold dilutions of ampicillin were used as a positive control. A bacterial cell suspension (prepared in the appropriate broth) of 100 μl, corresponding to 1 × 10^6 ^CFU/ml, was added in all wells except those in columns 10, 11 and 12, which served as saline, extract and media sterility controls, respectively. Controls for bacterial growth without plant extract were also included on each plate. The final concentration of bacteria in the assay was 5 × 10^5 ^CFU/ml. The final concentration of extracts ranged between 1000 to 7.8 μg/ml. Plates were then incubated at 37°C for 18 h overnight. After incubation, the MIC of each extract was determined as the lowest concentration at which no growth was observed in the duplicate wells. Twenty microliters of a p-iodonitro-tetrazolium violet solution (0.04%, w/v) (Sigma, USA) was then added to each well. The plates were incubated for a further 30 min, and estimated visually for any change in color from yellow to pink indicating reduction of the dye due to bacterial growth. The highest dilution (lowest concentration) that remained yellow corresponded to the MIC. Experiments were performed in duplicate.

#### Determination of anticancer activity-Cytotoxicity assay on human cancer cell lines

For the estimation of the *in vitro *cytotoxic potency of the investigated extracts, an established microtiter plate assay [9] was used with three human cancer cell lines: one lung cancer (A-427), one urinary bladder cancer (5637) and one breast cancer (MCF-7) line. Cell lines were obtained from the DMSZ, Braunschweig, Germany, and culture in RPMI 1640 medium with 10% FCS. Cytotoxicity determinations are based on cellular staining with crystal violet and were performed as previously described in detail. Briefly, a volume of 100 μl of a cell suspension was seeded into 96-well microliter plates at a density of 1000 cell/well. Twenty-four hours later, cells were treated with the plant extracts at five dilutions and exposed continuously to the extracts for the next 96 h. Etoposide was used as a positive control. At the end of the exposure time, the medium was removed and the cells were fixed with a glutaraldehyde solution. The cells were then stained with crystal violet and the optical density (OD) was measured at λ = 570 nm with a plate reader. The percent growth values were calculated by the following equation:

Growth (%) = OD_T _- OD_c, 0_/OD_c _- OD_c, 0 _× 100

Where OD_T _is the mean absorbance of the treated cells, OD_c _is the mean absorbance of the controls, OD_c, 0 _is the mean absorbance at the time the extract was added. The IC_50 _values were estimated by a linear least-squares regression of the growth values versus the logarithm of the extract concentration; only concentrations that yielded growth values between 10% and 90% were used in the calculation.

#### Determination of antioxidant activity (Scavenging Activity of DPPH Radical)

The DPPH free radical scavenging assay was carried out for the evaluation of the antioxidant activity. This assay measures the free radical scavenging capacity of the investigated extracts. DPPH is a molecule containing a stable free radical. In the presence of an antioxidant, which can donate an electron to DPPH, the purple colour typical for free DPPH radical decays, and the absorbance change at λ = 517 nm is measured. This test provides information on the ability of a compound to donate a hydrogen atom, on the number of electrons a given molecule can donate, and on the mechanism of antioxidant action. The method was carried out as previously described by [10]. The methanolic and aqueous extracts were redissolved in methanol and 5% ethanol, respectively, and various concentrations (10, 50, 100, 500 and 1000 μg/ml) of each extract were used. Similar concentrations of ascorbic acid were used as positive control. The assay mixture contained in a total volume of 1 ml, 500 μl of the extract, 125 μl prepared DPPH (1 mM in methanol) and 375 μl solvent (methanol or 5% ethanol). After 30 min incubation at 25°C, the decrease in absorbance was measured at λ = 517 nm. The radical scavenging activity was calculated from the equation:

% of radical scavenging activity = Abs_control _- Abs_sample_/Abs_control _× 100

#### Phytochemical screening of the methanolic extracts

The screening of chemical constituents was carried out with the methanol extracts by using chemical methods and thin-layer chromatography (TLC) using different mixtures of organic solvents as mobile phases. Several chemical reagents e.g. Dragendorf's reagent for alkaloids, borntraeger reagent for anthraquinons and etc. were used in the detection according to previously published methodology [11].

## Results

In the course of our screening for the antimicrobial, anticancer, and antioxidant and activities, a number of plants from different locations of the island Soqotra used in Yemeni traditional medicine were evaluated. A total of 52 extracts representing 26 plant species belonging to 17 families were submitted to biological screening. The botanical names, plant part used and the traditional uses of the plants in the collected areas are presented in Table 1.

### Antimicrobial activity

Table 2 shows the results of the antimicrobial activity of the investigated extracts in agar diffusion method. An inhibition zone > 15 mm was considered as a high antimicrobial activity. It was observed that the antimicrobial activity of the studied plant extracts was exhibited mainly against the Gram-positive bacteria. Consequently, the MIC-values were determined only against Gram-positive bacteria. The MIC values are reported in Table 3. In a general, among the investigated extracts the methanolic extracts exhibited the greatest antimicrobial effect.

**Table 2:** Antimicrobial activity of the investigated plants in agar diffusion test.

			Microbial strains tested						Multiresistant strains tested		
			
Plant species	Extracts	Extract yield in %	*S. a*.	*B. c*.	*M. f*.	*E. c*.	*P. e*.	*C. m*.	*S. e*. 847	*S. h*. 535	*S. a*. NGR
*Acacia pennivenia*	Methanolic Hot aqueous	12.18 5.97	22 20	12 -	22 20	16 -	14 -	- -	18 8	14 -	20 18
*Acanthospermum hispidum*	Methanolic Hot aqueous	16.14 7.22	16 12	- -	22 15	- -	- -	- -	18 8	12 -	20 12
*Acridocarpus socotranus*	Methanolic Hot aqueous	10.46 4.75	15 18	- -	16 16	- -	- -	- -	14 18	10 14	14 22
*Aloe perryi*	Methanolic Hot aqueous	20.27 18.82	- -	- -	- -	- -	- -	- -	18 -	10 -	20 14
*Ballochia atro-virgata*	Methanolic Hot aqueous	14.91 5.70	12 12	- -	- -	- -	- -	- -	10 -	- -	16 -
*Blepharis spiculifolia*	Methanolic Hot aqueous	5.04 5.51	12 -	- -	- -	- -	- -	- -	10 -	- -	16 -
*Boswellia dioscorides*	Methanolic Hot aqueous	40.45 14.31	18 11	16 8	20 12	8 -	10 -	10 -	28 28	18 16	38 28
*Boswellia socotrana*	Methanolic Hot aqueous	33.77 11.68	31 25	21 12	27 16	14 10	20 15	22 16	28 22	20 18	28 24
*Capparis cartilaginea*	Methanolic Hot aqueous	8.33 10.02	10 -	- -	- -	- -	- -	- -	- -	- -	- -
*Commiphora ornifolia*	Methanolic Hot aqueous	19.50 10.72	16 11	13 8	19 12	- -	- -	- -	18 18	14 10	18 20
*Corchorus erodioides*	Methanolic Hot aqueous	10.46 4.95	10 -	9 -	12 11	- -	- -	- -	- -	- -	- -
*Croton socotranus*	Methanolic Hot aqueous	14.91 6.78	10 -	8 -	15 -	- -	- -	- -	18 20	10 10	24 24
*Euclea divinorum*	Methanolic Hot aqueous	21.85 4.13	24 16	12 -	18 -	11 -	15 -	10 -	24 12	16 -	26 20
*Euphorbia socotrana*	Methanolic Hot aqueous	19.74 11.75	16 -	11 -	18 -	- -	- -	- -	20 20	12 12	26 26
*Eureiandra balfourii*	Methanolic Hot aqueous	11.60 8.58	10 12	- -	- 15	- -	- -	- -	- -	- -	- -
*Ficus cordata*	Methanolic Hot aqueous	9.06 10.50	10 16	- -	- -	- -	- -	- -	- -	- -	12 14
*Glossonema revoili*	Methanolic Hot aqueous	16.34 8.53	20 9	- -	- -	- -	- -	- -	- -	- -	- -
*Hibiscus noli-tangere*	Methanolic Hot aqueous	10.61 4.92	10 -	- -	- -	- -	- -	- -	- -	- -	- -
*Hypoestes pubescens*	Methanolic Hot aqueous	21.16 7.80	8 15	- -	- 14	- -	- -	15 12	- -	- -	- -
*Lannea transulta*	Methanolic Hot aqueous	8.04 9.15	13 14	- -	14 16	- -	- -	- 12	16 22	- 12	16 22
*Leucas samhaensis*	Methanolic Hot aqueous	11.47 5.25	- 19	- 14	20 24	- -	- -	- -	14 18	12 14	18 20
*Leucas virgata*	Methanolic Hot aqueous	14.11 3.37	18 17	11 -	17 15	- -	- -	- -	16 20	12 12	22 26
*Lycium sokotranum*	Methanolic Hot aqueous	10.93 5.05	20 17	- -	15 -	- -	- -	- -	12 -	12 -	20 20
*Maerua angolensis*	Methanolic Hot aqueous	22.01 13.80	- -	- -	- -	- -	- -	- -	- 12	- -	- 16
*Rhus thyrsiflora*	Methanolic Hot aqueous	5.88 11.50	20 20	12 12	18 21	- -	- -	- -	18 20	14 16	24 26
*Teucrium sokotranum*	Methanolic Hot aqueous	12.42 4.43	17 22	14 10	22 15	- -	- -	- -	14 18	10 14	16 20
Ampicillin 10 μg/disc Gentamicin 10 μg/disc Amphotericin 10 μg/disc			28 N.T. N.T.	24 N.T. N.T.	30 N.T. N.T.	N.T. 15 N.T.	N.T. 17 N.T.	N.T. N.T. 10	- N.T. N.T.	- N.T. N.T.	- N.T. N.T.

Only rarely do plants shown antimicrobial activity against Gram-negative bacteria and *Candida maltosa *except *Acacia pennivenia*, *Boswellia *species and *Euclea divinorum*. It was apparent that the multiresistant *Staphylococcus *strains demonstrate more sensitivity to the investigated extracts than the other antibiotic susceptible Gram-positive bacteria (Table 2). Moreover, it was found that with the exception of *Boswellia *species, *Euclea divinorum *and *Hypoestes pubescens *no extract showed antifungal activity against *Candida maltosa *(Table 2). The most pronounced activity with inhibition zones greater than 15 mm was found with the methanolic extracts of 10 plants (Table 2). The most effective plant was *Boswellia socotrana*, of which the methanolic and aqueous extracts demonstrated the greatest antimicrobial effect against all tested microorganisms (Table 2). The lowest MIC values were obtained against *Staphylococcus aureus *and *Micrococuss flavus *by the methanolic extracts of *Boswellia *species (125 μg/ml) (Table 3).

**Table 3:** Free radical scavenging activity and MIC of the investigated plants.

Plant species	Extracts	Radical scavenging activity in %					MIC in μg/ml		
		
		10 (μg/ml)	50 (μg/ml)	100 (μg/ml)	500 (μg/ml)	1000 (μg/ml)	*S. aureus*	*B. subtilis*	*M. flavus*
*Acacia pennivenia*	Methanolic Hot aqueous	25.4 4.8	78.5 29.2	88.9 31.1	95.0 42.8	94.0 67.0	250 500	500 1000	250 500
*Acanthospermum hispidum*	Methanolic Hot aqueous	2.8 5.2	13.8 11.6	25.9 14.4	55.5 26.3	94.7 27.4	500 > 1000	1000 > 1000	500 > 1000
*Acridocarpus socotranus*	Methanolic Hot aqueous	5.1 0.6	40.8 6.7	80.9 37.6	92.6 46.5	94.6 45.3	250 250	1000 > 1000	250 250
*Aloe perryi*	Methanolic Hot aqueous	6.7 2.1	19.8 6.4	28.7 20.8	92.4 36.4	94.1 45.9	> 1000 > 1000	> 1000 > 1000	> 1000 > 1000
*Ballochia atro-virgata*	Methanolic Hot aqueous	2.1 2.0	5.3 2.7	45.7 34.5	56.8 43.7	94.2 36.7	> 1000 > 1000	> 1000 > 1000	> 1000 > 1000
*Blepharis spiculifolia*	Methanolic Hot aqueous	14.8 1.6	13.3 4.4	25.1 34.3	64.6 38.3	91.5 46.8	> 1000 > 1000	> 1000 > 1000	> 1000 > 1000
*Boswellia dioscorides*	Methanolic Hot aqueous	25.8 7,8	89.8 39.0	96.1 65.9	96.3 68.2	96.8 74.6	125 500	500 1000	125 500
*Boswellia socotrana*	Methanolic Hot aqueous	26.1 2.9	88.2 30.9	94.6 58.5	94.9 62.1	95.1 75.8	125 250	250 500	125 250
*Capparis cartilaginea*	Methanolic Hot aqueous	0.2 3.1	3.8 1.1	2.1 22.8	51.2 27.5	87.6 28.2	> 1000 > 1000	> 1000 > 1000	> 1000 > 1000
*Commiphora ornifolia*	Methanolic Hot aqueous	20.8 10.6	85.9 25.2	95.0 49.1	95.4 66.2	95.2 72.9	250 500	500 1000	250 500
*Corchorus erodioides*	Methanolic Hot aqueous	10.4 4.2	14.3 5.1	27.6 37.6	95.1 57.9	94.1 59.7	> 1000 > 1000	> 1000 > 1000	> 1000 > 1000
*Croton socotranus*	Methanolic Hot aqueous	5.3 2.0	12.6 2.9	22.2 10.1	78.9 23.5	93.1 26.4	500 > 1000	1000 > 1000	500 > 1000
*Euclea divinorum*	Methanolic Hot aqueous	3.1 14.3	10.6 27.1	36.2 41.5	93.1 52.6	88.6 56.1	250 > 1000	1000 > 1000	500 > 1000
*Euphorbia socotrana*	Methanolic Hot aqueous	16.7 1.9	67.4 8.8	95.5 28.7	96.3 36.2	95.2 40.1	500 > 1000	1000 > 1000	500 > 1000
*Eureiandra balfourii*	Methanolic Hot aqueous	15.0 7.3	17.8 23.0	8.3 35.4	19.1 42.7	57.7 41.0	> 1000 > 1000	> 1000 > 1000	> 1000 > 1000
*Ficus cordata*	Methanolic Hot aqueous	2.5 1.6	12.5 9.3	31.0 16.4	89.2 18.9	91.6 20.4	> 1000 > 1000	> 1000 > 1000	> 1000 > 1000
*Glossonema revoili*	Methanolic Hot aqueous	1.7 0.3	14.9 2.7	26.8 2.5	38.6 7.8	76.4 8.1	> 1000 > 1000	> 1000 > 1000	> 1000 > 1000
*Hibiscus noli-tangere*	Methanolic Hot aqueous	0.6 1.4	7.3 0.8	18.4 2.8	85.9 5.6	89.0 6.6	> 1000 > 1000	> 1000 > 1000	> 1000 > 1000
*Hypoestes pubescens*	Methanolic Hot aqueous	1.5 2.6	4.0 4.2	16.6 3.1	38.7 16.1	72.6 18.3	> 1000 1000	> 1000 > 1000	> 1000 500
*Lannea transulta*	Methanolic Hot aqueous	21.9 36.7	64.2 33.2	95.4 37.2	92.1 32.3	98.6 29.9	1000 1000	> 1000 > 1000	1000 1000
*Leucas samhaensis*	Methanolic Hot aqueous	2.3 3.8	9.6 5.6	8.0 37.3	61.7 49.3	88.8 47.1	> 1000 500	> 1000 1000	500 500
*Leucas virgata*	Methanolic Hot aqueous	1.5 26.2	1.1 34.8	2.8 39.5	27.6 44.0	81.9 51.9	500 500	> 1000 > 1000	1000 500
*Lycium sokotranum*	Methanolic Hot aqueous	5.6 26.5	27.8 30.3	29.8 31.2	91.4 37.4	94.2 39.5	500 1000	1000 > 1000	500 > 1000
*Maerua angolensis*	Methanolic Hot aqueous	13.8 29.1	19.3 65.1	32.0 69.1	52.5 68.3	82.3 73.9	> 1000 > 1000	> 1000 > 1000	> 1000 > 1000
*Rhus thyrsiflora*	Methanolic Hot aqueous	3.8 1.1	29.4 9.3	55.9 12.8	95.6 22.4	95.4 30.5	500 500	> 1000 1000	500 500
*Teucrium sokotranum*	Methanolic Hot aqueous	3.4 24.2	2.8 28.7	16.4 38.2	41.0 47.6	92.1 61.4	500 250	1000 1000	500 500
Ascorbic acid		42.8	94.2	96.8	96.6	96.9			

### *In vitro *anticancer activity

Table 4 presents the IC_50 _values for the in vitro cytotoxic activity of the investigated 26 methanolic extracts. From our experience, the most aqueous extracts don't show any notable in vitro anticancer effect at the highest concentration tested (50 μg/ml). Generally, the aqueous extracts may exhibit only a weak cytotoxic effect starting with a concentration of 250 μg/ml. Thus, these extracts were excluded in our screen. The results in Table 4 demonstrate that out of the 26 investigated plants (only methanolic extracts) three plant extracts had noteworthy cytotoxic effects in all three cell lines tested, namely: *Ballochia atro-virgata *(IC_50 _between 1.5 and 2.8 μg/ml), *Eureiandra balfourii *(IC_50 _between 0.8 and 3.4 μg/ml) and *Hypoestes pubescens *(IC_50 _between 4.8 and 8.2 μg/ml) (Table 4). Moreover, the extracts of *Acanthospermum hispidum*, *Boswellia dioscorides*, *Boswellia socotrana*, *Commiphora ornifolia *and *Euphorbia socotrana *exhibited a pronounced cytotoxic effect against all tested cell lines ((IC_50 _between 9.3 and 38.5 μg/ml). The other plant extracts demonstrated no significant cytotoxic effect at the highest tested concentration of 50 μg/ml.

**Table 4:** IC_50 _values (μg/ml) for cell growth inhibition of the investigated methanolic extracts on three human cancer cell lines and phytochemical screening.

Plant species	Extracts	Cell lines			Phytochemical screening
			
		5637	MCF-7	A-427	
*Acacia pennivenia*	Methanolic	> 50	> 50	> 50	Saponins, tannins
*Acanthospermum hispidum*	Methanolic	9.37 ± 1.98	19.92 ± 8.94	16.70 ± 2.32	Sesquiterpene lactones
*Acridocarpus socotranus*	Methanolic	> 50	> 50	> 50	Flavonoids, terpenoids
*Aloe perryi*	Methanolic	> 50	> 50	> 50	Anthraquinons, flavonoids
*Ballochia atro-virgata*	Methanolic	2.83 ± 2.06	2.94 ± 0.28	1.58 ± 0.54	Terpenoids
*Blepharis spiculifolia*	Methanolic	> 50	> 50	> 50	Phenolic compounds, terpenoids
*Boswellia dioscorides*	Methanolic	29.0 ± 8.26	> 50	27.60 ± 10.31	Volatile oil, terpenoids, flavonoids
*Boswellia socotrana*	Methanolic	18.4 ± 4.66	> 50	24.9 ± 8.73	Volatile oil, terpenoids, flavonoids
*Capparis cartilaginea*	Methanolic	> 50	> 50	> 50	Glucosinolates, flavonoids
*Commiphora ornifolia*	Methanolic	30.2 ± 3.12	38.5 ± 1.93	35.1 ± 3.15	Volatile oil, terpenoids, flavonoids
*Corchorus erodioides*	Methanolic	> 50	> 50	> 50	Flavonoids, phenolic compounds
*Croton socotranus*	Methanolic	> 50	> 50	> 50	Flavonoids, terpenoids, tannins
*Euclea divinorum*	Methanolic	> 50	> 50	> 50	Phenolic acids, tannins
*Euphorbia socotrana*	Methanolic	42.0 ± 7.43	> 50	38.1 ± 13.49	Terpenoids, flavonoids, steroids, tannins
*Eureiandra balfourii*	Methanolic	0.82 ± 0.15	3.47 ± 1.18	2.25 ± 0.57	Trepenoids, flavonoids
*Ficus cordata*	Methanolic	> 50	> 50	> 50	Tannins, terpenoids
*Glossonema revoili*	Methanolic	> 50	> 50	> 50	Steroids, flavonoids
*Hibiscus noli-tangere*	Methanolic	> 50	> 50	> 50	Tannins, lignans
*Hypoestes pubescens*	Methanolic	8.25 ± 3.49	7.03 ± 2.02	4.85 ± 1.70	Alkaloids, terpenoids
*Lannea transulta*	Methanolic	> 50	> 50	> 50	Tannins, flavonoids
*Leucas samhaensis*	Methanolic	> 50	> 50	> 50	Volatile oil, terpenoids and flavonoids
*Leucas virgata*	Methanolic	> 50	> 50	> 50	Volatile oil, terpenoids and flavonoids
*Lycium sokotranum*	Methanolic	> 50	> 50	> 50	Alkaloids
*Maerua angolensis*	Methanolic	> 50	> 50	> 50	Glucosinolates, tannins
*Rhus thyrsiflora*	Methanolic	> 50	> 50	> 50	Flavonoids, terpenoids, tannins
*Teucrium sokotranum*	Methanolic	> 50	> 50	> 50	Volatile oil, terpenoids
Etoposide (μM)		0.54 ± 0.30	0.50 ± 0.19	0.13 ± 0.10	

Values are averages and standard deviations of three or more independent determinations.

### Radical scavenging activity

The methanol extracts of seven plants, namely: *Acacia pennivenia*, *Acridocarpus socotranus*, *Boswellia *dioscorides, *Boswellia socotrana*, *Commiphora ornifolia*, *Euphorbia socotrana*, and *Lannea transulta *showed a high effective free radical scavenging in the DPPH assay (Table 3). These extracts exhibited a noticeable antioxidant effect at low concentrations (Table 3). So the methanolic extracts of *Acacia pennivenia*, *Boswellia dioscorides*, *Boswellia socotrana *and *Commiphora ornifolia *exhibited a great antioxidant effect at 50 μg/ml (78, 89, 88% and 85%, respectively), comparing with the effect of ascorbic acid at this concentration (Table 3). The hot aqueous extracts of all investigated plants showed only weak antioxidant effect (Table 3).

### Phytochemical screening

The results of the phytochemical screening of the investigated methanolic extracts indicated the presence of different types of active constituents like flavonoids, terpenoids, tannins, volatile oils, etc... (Table 4).

## Discussion

In continuation of our search for substances of plant origin with pharmacological effects, we have screened 26 plants collected from the island Soqotra, Yemen for their antimicrobial, cytotoxic and antioxidant activities and for their chemical content. It is important to mention that this work represents the first report on the antimicrobial, cytotoxic and antioxidant activities of extracts from 20 endemic plants (Table 1). The existing knowledge about the other six investigated plants is in many cases very limited.

A correlation was found between the antibacterial activity observed by agar diffusion assay and MIC determination. It is interesting to note that these plant extracts showed more activity on multi-drug resistant *Staphylococcus *strains than the antibiotic susceptible Gram-positive bacteria.

It was demonstrated previously that alcoholic extract of different species of *Acacia *like *A. arabica*, *A. nilotica *and *A. auriculiformis *have antibacterial activity [12-15], and that the isolated saponines from *A. auriculiformis *were responsible for the noticed effect [15]. Previous work indicated that both bark and heartwood extracts of *A. confusa *clearly have strong antioxidant effects [16]. Phenolic compounds were isolated from the bark of *A. confuse*, which were mainly responsible for the antioxidant effect of this plant [17]. Furthermore, triterpenoid saponins were isolated from *A. victoriae*, which induced decreased tumor cell proliferation and induced apoptosis [18]. In addition, a significant reduction in the values of tumor burden and tumor incidence was observed in mice treated by oral gavage with the *A. nilotica *gum and leaf extracts [19]. The antimicrobial and antioxidant effects we have found with extracts from *A. pennivenia *are in accordance with these data. The phytochemical screening revealed the presence of saponins and phenolic compounds like tannins in the methanolic extract of *A. pennivenia*, which could be responsible for these activities. On the other hand, our results of cytotoxic activity were not in agreement with the anticancer effect noted with other *Acacia *species; i.e., at the highest concentration used in our screening (50 μg/ml), the extracts of *A. pennivenia *exhibit no cytotoxic activity.

The antimicrobial effect of leaves and flowers extracts of *Acanthospermum hispidum *has been previously investigated [20]. Moreover, the isolation of sesquiterpene lactones was reported [21,22]. Our screening results confirmed the antimicrobial effect of the alcoholic extract. The hot aqueous extract exhibited a moderate antibacterial effect in contrast with data presented earlier [20]. In addition, the methanolic extract brought about a pronounced growth inhibitory effect against all tested cancer cell lines we tested it against. These effects are most likely due to the presence of sequiterpene lactones identified in our phytochemical screening and in literature data.

Whereas the investigation of *Acridocarpus vivy *showed cytotoxic effect against some cancer cell lines [23], the methanolic extract of *Acridocarpus socotranus *demonstrated no cytotoxic activity in our screen. However the plant extract exhibited a strong antioxidant and moderate antibacterial effects. This finding may be correlated with the presence of flavonoids and terpenoids.

In contrast to Ali and co-workers [24], who found a promising antimicrobial effect for *Aloe perryi*, our extracts of *Aloe perryi *only showed activity against multiresistant bacteria. Although the phytochemical screening illustrated the presence of anthraquinons, which are mostly responsible for cytotoxic and antioxidant activity [25], our results do not indicat any cytotoxic activity at the highest concentration tested (50 μg/ml) and exhibited a moderate antioxidant effect only at high concentrations (500 and 1000 μg/ml)

It is important to consider that we found no published data on the genus *Ballochia *and *Eureiandra*. So this is the first report on pharmacological and chemical investigation of plants of these genera. The remarkable cytotoxic effect of both plants with IC_50 _values between 0.8 and 3.4 μg/ml is significant. According to the criteria of the American National Cancer Institute, 30 μg/ml is the upper IC_50 _limit considered promising for purification of a crude extract [26]. Therefore, the highest concentration tested (50 μg/ml) in our screening was slightly above this limit.

The chemical screening showed the presence of terpenoids in both plants (mainly diterpenoids and triterpenoids), which could be correlated with this effect. *E*.*balfourii *belongs to the family cucurbitaceae, plants of which are famous for their cytotoxic tetracyclic triterpenoids known as cucurbitacins [27-29]. This type of compounds could be responsible for the observed activity in *E*.*balfourii*.

In our previous studies, the antimicrobial effects of some *Boswellia *species namely *B. elongata *and *B. ameero *were investigated [3]. It was found that both plants exhibited a strong antimicrobial effect only against Gram positive bacteria. Both plants demonstrated no cell growth inhibition on five human cancer cell lines [6]. Furthermore, it was reported that the extract of *B. serrata *showed an effect against some bacterial strains [30]. The antimicrobial activity and the chemical content of the volatile oil of some *Boswellia *species like *B. carteri*, *B. papyrifera*, *B. serrata *and *B. rivae *were determined [31]. It was shown that the oils consisted of several monoterpenes, sesquiterpenes and diterpenes are responsible for the effect observed. The *B. socotrana *and B. *dioscorides *we investigated produced extracts with the most effective activity against all tested microorganisms. The essential oils and the terpenoids determined in our phytochemical screening are mostly responsible for this effect. Besides this noticeable antimicrobial effect, the methanolic extracts manifested a considerable cytotoxic effect against two cell lines (5637 and A-427) with IC_50 _values between 18 and 29 μg/ml. For this effect, boswellic acids, which may represent a considerable part of the chemical content, could contribute to this observed effect. Furthermore, both plants demonstrated even at low concentration (50 μg/ml) a remarkable radical scavenging effect (88 and 89%). This effect may be attributed to the present flavonoids.

Among the most interesting plants, *Commiphora ornifolia *showed similar antimicrobial activities with *Boswellia *plants. The same extract manifested a high antioxidant effect (85%) at 50 μg/ml. The extracts and isolated compounds from *Commiphora opobalsamum *exhibited similar antimicrobial and antioxidant activities [32]. Thus, the estimated antimicrobial and antioxidant effects of our investigated *C. ornifolia *are in accordance with these data. Whereas no cytotoxic activity in *C. myrrha *was found [33], we observed an antiproliferative effect of the methanolic extract against all three cell lines with IC_50 _values between 30 and 38 μg/ml. The determined effects of *C. ornifolia *are mostly attributed to the essential oil, flavonoids and tritepenes found in the methanolic extract.

Another interesting plant was *Euphorbia socotrana*, which demonstrated considerable antimicrobial, cytotoxic and antioxidant activities. Our data are in agreement with literature data of other *Euphorbia *species as *E*.*thymifolia*, *E*.*hirta *and E. *cheiradenia *[34-36]. Studies have demonstrated that some triterpenoids isolated from *Euphorbia *species are responsible for the antimicrobial effect and that some of these compounds were able to induce moderate apoptosis in at least one cancer cell line [37,38]. The presence of such terpenoids in our *E. socotrana *may explain the biological effects seen in our screens.

Some authors reported on investigation with some *Hypoestes *species, where the isolation of two cell growth inhibitory phenanthroindolizidine alkaloids termed hypoestestatin 1 and hypoestestatin 2 from the East African shrub *H*.*verticillaris *was described [39]. In addition, several antifungal diterpenoids from *H*.*serpens *were isolated [40]. The methanolic extract of *H*. *pubescens *tested in our screens showed a remarkable antifungal effect against *Candida maltosa *and an extraordinary cytotoxic effect against all tested cancer cell lines. Apparently these observed activities are correlated with the presence of the terpenoids and alkaloids found in the phytochemical screening.

The notably antimicrobial effect of *Rhus thyrsiflora *and *Teucrium sokotranum *was consistent with literature data of other *Rhus *and *Teucrium *species [41,42]. On the other hand, other authors noted a strong antioxidant effect of some *Teucrium *species and *R*.*coriaria*, which is not supported by results obtained in our screening [43,44]. Moreover, a cytotoxic activity for the sap of the lacquer tree *R. succedanea *was described previously [45], whereas the investigated *R. thyrsiflora *showed no growth inhibitory effect on the tested cancer cell lines.

## Conclusion

In conclusion, the results in the present study are agreed to some extent with the traditional uses of the plants investigated. Our results further support the idea that medicinal plants can be promising sources of potential anticancer and antimicrobial agents and antioxidants. The present results will form the basis for selection of plant species for further investigation in the potential discovery of new natural bioactive compounds. Studies aimed at the isolation and structure elucidation of anticancer, antibacterial and antioxidant active constituents from some plants e.g. *Acacia pennivenia*, *Ballochia atro-virgata*, *Boswellia dioscorides*, *Boswellia socotrana*, *Commiphora ornifolia*, *Euphorbia socotrana*, *Eureiandra balfourii *and *Hypoestes pubescens *are in progress.

## Competing interests

The authors declare that they have no competing interests.

## Authors' contributions

RAM carried out the study design, plant collection, experimental work, data collection and interpretaion, literature search and manuscript preparation. RG provided assistance in evaluation of the anticancer activity and data interpretaion. UL and PJB supervised the work, evaluated the data and corrected the manuscript for publication. All authors read and approved the final manuscript.

## Pre-publication history

The pre-publication history for this paper can be accessed here:

http://www.biomedcentral.com/1472-6882/9/7/prepub

## References

[B1] Newman DJ Cragg GM Snader KM Natural products as sources of new drugs over the period 1981–2002 J Nat Prod 2003 66 022 1037 10.1021/np030096l 12880330

[B2] Miller GA Morris M Ethnoflora of the Soqotra Archipelago 2004 The Royal Botanic Garden Edinburgh, UK: Printed by the Charlesworth Group, Huddersfield, UK

[B3] Mothana RAA Lindequist U Antimicrobial activity of some medicinal plants of the island Soqotra J Ethnopharmacol 2005 96 177 181 10.1016/j.jep.2004.09.006 15588668

[B4] Mothana RAA Mentel R Reiss C Lindequist U Phytochemical screening and antiviral activity of some medicinal plants of the island soqotra Phytother Res 2006 20 298 302 10.1002/ptr.1858 16557613

[B5] Oleski A Lindequist U Mothana RA Melzig MF Screening of selected Arabian medicinal plant extracts for inhibitory activity against peptidases Pharmazie 2006 61 4 359 361 16649556

[B6] Mothana RAA Gruenert R Lindequist U Bednarski PJ Study of the anticancer potential of Yemeni plants used in folk medicine Pharmazie 2007 62 305 307 17484289

[B7] Bauer Aw Kirby WMM Sheriss JC Turck M Antibiotic susceptibility testing by standardized single disk method Americ J Clinic Pathol 1966 45 493 496 5325707

[B8] Mann CM Markham JL A new method for determining the minimum inhibitory concentration of essential oils J Appl Microbiol 1998 84 538 544 10.1046/j.1365-2672.1998.00379.x 9633651

[B9] Bracht K Boubakari R Grünert R Bednarski PJ Correlations between the activities of 19 antitumor agents and the intracellular GSH concentrations in a panel of 14 human cancer cell lines: Comparisons with the NCI data Anti-Cancer Drugs 2006 17 41 51 10.1097/01.cad.0000190280.60005.05 16317289

[B10] Brand WW Cuvelier HE Berset C Use of a free radical method to evaluate antioxidant activity Food Sci Technol 1995 82 25 30

[B11] Wagner H Bladt S Plants Drug Analysis: A Thin Layer Chromatography Atlas 1996 2 Berlin: Springer 306 364

[B12] Almas K The antimicrobial effects of seven different types of Asian chewing sticks Odontostomatol Trop 2001 24 96 17 20 11887585

[B13] Al-Fatimi M Wurster M Schroeder G Lindequist U Antioxidant, antimicrobial and cytotoxic activities of selected medicinal plants from Yemen J Ethnopharmacol 2007 111 3 657 666 10.1016/j.jep.2007.01.018 17306942

[B14] Rani P Khullar N Antimicrobial evaluation of some medicinal plants for their anti-enteric potential against multi-drug resistant *Salmonella typhi* Phytother Res 2004 18 8 670 673 10.1002/ptr.1522 15476301

[B15] Mandal P Sinha Babu SP Mandal NC Antimicrobial activity of saponins from *Acacia auriculiformis* Fitoterapia 2005 76 5 462 465 10.1016/j.fitote.2005.03.004 15951137

[B16] Chang ST Wu JH Wang SY Kang PL Yang NS Shyur LF Antioxidant activity of extracts from *Acacia confusa *bark and heartwood J Agric Food Chem 2001 49 7 3420 3424 10.1021/jf0100907 11453785

[B17] Tung YT Wu JH Kuo YH Chang ST Antioxidant activities of natural phenolic compounds from *Acacia confusa *bark Bioresour Technol 2007 98 5 1120 1123 10.1016/j.biortech.2006.04.017 16766181

[B18] Mujoo K Haridas V Hoffmann JJ Waechter GA Hutter LK Lu Y Blake ME Jayatilake GS Bailey D Mills GB Gutterman JU Triterpenoid saponins from *Acacia victoriae *(Bentham) decrease tumor cell proliferation and induce apoptosis Cancer Res 2001 15;61 14 5486 5490 11454696

[B19] Meena PD Kaushik P Shukla S Soni AK Kumar M Kumar A Anticancer and antimutagenic properties of Acacia nilotica (Linn.) on 7,12-dimethylbenz(a)anthracene-induced skin papillomagenesis in Swiss albino mice Asian Pac J Cancer Prev 2006 7 4 627 632 17250441

[B20] Fleischer TC Ameade EP Sawer IK Antimicrobial activity of the leaves and flowering tops of *Acanthospermum hispidum* Fitoterapia 2003 74 1–2 130 132 10.1016/S0367-326X(02)00290-3 12628407

[B21] Jakupovic J Baruah RN Bohlmann F Msonthi JD Further Acanthospermolides from *Acanthospermum hispidum* Planta Medica 1986 52 2 154 155 10.1055/s-2007-96910417345222

[B22] Cartagena E Bardon A Catalan CA de Hernandez ZN Hernandez LR Joseph-Nathan P Germacranolides and a new type of guaianolide from *Acanthospermum hispidum* J Nat Prod 2000 63 10 1323 1328 10.1021/np9905057 11076545

[B23] Cao S Guza RC Miller JS Andriantsiferana R Rasamison VE Kingston DG Cytotoxic triterpenoids from *Acridocarpus vivy *from the Madagascar rain forest J Nat Prod 2004 67 6 986 989 10.1021/np040058h 15217279

[B24] Ali NA Jülich WD Kusnick C Lindequist U Screening of Yemeni medicinal plants for antibacterial and cytotoxic activities J Ethnopharmacol 2001 74 2 173 179 10.1016/S0378-8741(00)00364-0 11167035

[B25] Niciforovic A Adzic M Spasic SD Radojcic MB Antitumor effects of a natural anthracycline analog (Aloin) involve altered activity of antioxidant enzymes in HeLaS3 cells Cancer Biol Ther 2007 6 8 1200 1205 17726368 10.4161/cbt.6.8.4383

[B26] Suffness M Pezzuto JM Hostettmann K Assays related to cancer drug discovery Methods in Plant Biochemistry: Assays for Bioactivity 1990 London: 6. Academic Press 71 133

[B27] Jayaprakasam B Seeram NP Nair MG Anticancer and antiinflammatory activities of cucurbitacins from *Cucurbita andreana* Cancer Lett 2003 189 11 16 10.1016/S0304-3835(02)00497-4 12445672

[B28] Chen J Tian R Qiu M Lu L Zheng Y Zhang Z Trinorcucurbitane and cucurbitane triterpenoids from the roots of *Momordica charantia* Phytochemistry 2008 69 1043 1048 10.1016/j.phytochem.2007.10.020 18045630

[B29] Wang D-C Xiang H Li D Gao H-Y Cai H Wu L-J Deng X-M Purine-containing cucurbitane triterpenoids from *Cucurbita pepo *cv dayangua Phytochemistry 2008 69 1434 1438 10.1016/j.phytochem.2008.01.019 18325551

[B30] Weckesser S Engel K Simon-Haarhaus B Wittmer A Pelz K Schempp CM Screening of plant extracts for antimicrobial activity against bacteria and yeasts with dermatological relevance Phytomedicine 2007 14 7–8 508 516 10.1016/j.phymed.2006.12.013 17291738

[B31] Camarda L Dayton T Di Stefano V Pitonzo R Schillaci D Chemical composition and antimicrobial activity of some oleogum resin essential oils from *Boswellia *spp. (Burseraceae) Ann Chim 2007 97 9 837 844 10.1002/adic.200790068 17970299

[B32] Abbas FA Al-Massarany SM Khan S Al-Howiriny TA Mossa JS Abourashed EA Phytochemical and biological studies on Saudi *Commiphora opobalsamum *L Nat Prod Res 2007 21 5 383 391 10.1080/14786410600942025 17487607

[B33] Shoemaker M Hamilton B Dairkee SH Cohen I Campbell MJ In vitro anticancer activity of twelve Chinese medicinal herbs Phytother Res 2005 19 7 649 651 10.1002/ptr.1702 16161030

[B34] Lin CC Cheng HY Yang CM Lin TC Antioxidant and antiviral activities of *Euphorbia thymifolia *L J Biomed Sci 2002 9 6 Pt 2 656 664 12432232 10.1159/000067281

[B35] Amirghofran Z Bahmani M Azadmehr A Javidnia K Induction of apoptosis in leukemia cell lines by *Linum persicum *and *Euphorbia cheiradenia* J Cancer Res Clin Oncol 2006 132 7 427 432 10.1007/s00432-006-0084-x 16477442 PMC12161081

[B36] Sudhakar M Rao ChV Rao PM Raju DB Venkateswarlu Y Antimicrobial activity of *Caesalpinia pulcherrima*, *Euphorbia hirta *and *Asystasia gangeticum* Fitoterapia 2006 77 5 378 380 10.1016/j.fitote.2006.02.011 16730921

[B37] Madureira AM Ascenso JR Valdeira L Duarte A Frade JP Freitas G Ferreira MJ Evaluation of the antiviral and antimicrobial activities of triterpenes isolated from *Euphorbia segetalis* Nat Prod Res 2003 17 5 375 380 10.1080/14786410310001605841 14526920

[B38] Madureira AM Spengler G Molnar A Varga A Molnar J Abreu PM Ferreira MJ Effect of cycloartanes on reversal of multidrug resistance and apoptosis induction on mouse lymphoma cells Anticancer Res 2004 24 2B 859 864 15161038

[B39] Pettit GR Goswami A Cragg GM Schmidt JM Zou JC Antineoplastic agents, 103. The isolation and structure of hypoestestatins 1 and 2 from the East African *Hypoestes verticillaris* J Nat Prod 1984 47 6 913 919 10.1021/np50036a001 6533268

[B40] Rasoamiaranjanahary L Guilet D Marston A Randimbivololona F Hostettmann K Antifungal isopimaranes from *Hypoestes serpens* Phytochemistry 2003 64 2 543 548 10.1016/S0031-9422(03)00154-7 12943772

[B41] Saxena G McCutcheon AR Farmer S Towers GH Hancock RE Antimicrobial constituents of *Rhus glabra* J Ethnopharmacol 1994 42 2 95 99 10.1016/0378-8741(94)90102-3 8072309

[B42] Ricci D Fraternale D Giamperi L Bucchini A Epifano F Burini G Curini M Chemical composition, antimicrobial and antioxidant activity of the essential oil of *Teucrium marum *(Lamiaceae) J Ethnopharmacol 2005 98 1–2 195 200 10.1016/j.jep.2005.01.022 15763383

[B43] Ozcan M Antioxidant activities of rosemary, sage, and sumac extracts and their combinations on stability of natural peanut oil J Med Food 2003 6 3 267 270 10.1089/10966200360716698 14585194

[B44] Kadifkova Panovska T Kulevanova S Stefova M In vitro antioxidant activity of some *Teucrium *species (Lamiaceae) Acta Pharm 2005 55 2 207 214 16179134

[B45] Huang CP Fang WH Lin LI Chiou RY Kan LS Chi NH Chen YR Lin TY Lin SB Anticancer activity of botanical alkyl hydroquinones attributed to topoisomerase II poisoning Toxicol Appl Pharmacol 2008 227 3 331 33 10.1016/j.taap.2007.11.014 18164362

